# Quality of Water and Antibiotic Resistance of *Escherichia coli* from Water Sources of Hilly Tribal Villages with and without Integrated Watershed Management—A One Year Prospective Study

**DOI:** 10.3390/ijerph110606156

**Published:** 2014-06-12

**Authors:** Sandeep S. Nerkar, Ashok J. Tamhankar, Smita U. Khedkar, Cecilia Stålsby Lundborg

**Affiliations:** 1 Department of Public Health Sciences, Global Health (IHCAR), Karolinska Institutet, Stockholm, SE 17177, Sweden; E-Mails: ejetee@gmail.com (A.J.T.); Cecilia.Stalsby.Lundborg@ki.se (C.S.L.); 2 Department of Environmental Medicine, Indian Initiative for Management of Antibiotic Resistance, R. D. Gardi Medical College, Ujjain, 456010, India; 3 Bac-test Laboratory, College Road, Nashik, 422005, India; E-Mail: udayk_nsk@sancharnet.in

**Keywords:** integrated watershed management, hilly tribal region, water quality, *E. coli* contamination, antibiotic resistance

## Abstract

In many hilly tribal areas of the world, water scarcity is a major problem and diarrhoea is common. Poor quality of water also affects the environment. An integrated watershed management programme (IWMP) aims to increase availability of water and to improve life conditions. Globally, there is a lack of information on water contamination, occurrence of diarrhoea and antibiotic resistance, a serious global concern, in relation to IWMP in hilly tribal areas. Therefore, a prospective observational study was conducted during 2011–2012 in six villages in a hilly tribal belt of India, three with and three without implementation of an IWMP, to explore quality of water, diarrhoeal cases in the community and antibiotic resistance of *Escherichia coli* from water sources. The results showed that physico-chemical quality of water was within limits of safe consumption in all samples. The odds of coliform contamination in water samples was 2.3 times higher in non-watershed management villages (NWMV) compared to integrated watershed management villages (IWMV) (95% CI 0.8–6.45, *p* = 0.081). The number of diarrhoeal cases (18/663 *vs.* 42/639, *p* < 0.05) was lower in IWMV as compared to NWMV. Overall *E. coli* isolates showed high susceptibility to antibiotics. Resistance to a wider range of antibiotics was observed in NWMV.

## 1. Introduction

In many parts of the world, including India, tribal people live in hilly terrain [1]. Water scarcity is their main concern and contamination of water and diarrhoeal cases are common. Less availability and poor quality of water affects the environment as well as various life conditions. Open dug wells are most often the source of water in these tribal areas [2] and such wells are prone to physico-chemical and microbial contamination. This poses a risk to human health. Bacteria are often responsible for water contamination and subsequent disease. Antibiotics are used in the control of bacterial infections, but are also commonly unnecessarily used for non-bacterial infections. Development of resistance by bacteria makes antibiotics ineffective for those in need of treatment [3] and this is now of serious global concern. Further, water contaminated by resistant bacteria poses the risk of spread of antibiotic resistance in the environment [4].

A watershed is an area or a region which contributes rainfall water to a river or lake. Watershed management, if implemented effectively, can help to solve the scarcity of water in that area. Classically, watershed management mainly focuses on soil and water conservation with an aim to increase availability of water [5]. The modern concept of integrated watershed management (IWM), however, besides soil and water conservation, also includes plantation of trees, improving agriculture, improving hygiene and sanitation, formation of self-help groups and proper utilization and management of available natural resources [6]. 

Globally, there is a lacuna of information on water contamination and antibiotic resistance in relation to watershed management, particularly in hilly tribal areas. Therefore this study aimed to explore the quality of water, occurrence of bacterial contamination in water, incidence of diarrhoeal cases, antibiotic use in the community and antibiotic resistance of *Escherichia coli* found in the water sources of hilly tribal villages in the context of integrated watershed management. *E. coli* was selected as an indicator bacterial species, as it is commonly used to study the bacterial contamination in water and also for studying antibiotic resistance patterns [7].

## 2. Experimental Section

### 2.1. Study Setting and Design

This prospective observational study was conducted over a period of one year from July 2011 to June 2012 in a tribal belt of Thane district of Maharashtra state, India. The rainy season occurs in this area during June to September and the remaining months is the dry season. The terrain in this area is hilly and the area is characterized by scarcity of water, except for during the rainy season. This suggests a need for conservation and proper management of water. Six tribal villages located within a radius of 15 km were chosen purposively for the study. In one set of three villages, an integrated watershed management programme (IWMP) had already been implemented. The components of the implemented IWMP were soil and water conservation, plantation of trees, improvement in agricultural and livestock production, awareness programme on hygiene and sanitation, formation of self-help groups and community participation in all developmental activities. These villages are hereafter referred to as integrated watershed management villages (IWMV). The remaining three villages, hereafter referred to as non-watershed management villages (NWMV), were located in an adjoining watershed, where no watershed management programme had been implemented.

Open dug community wells were the major source of water in both IWMV and NWMV. All together a total of seven such wells were identified and labeled. The four wells of IWMV were labeled IWMV1 to IWMV4, whereas the three wells of NWMV were labeled NWMV1 to NWMV3. 

Two water samples, both of 250 mL were collected from each well at monthly interval. The samples were collected in sterilized glass bottles following standard procedures [8]. Immediately after collection, the first sample was evaluated using a field level rapid testing kit (WT023, Hi-Media, Mumbai, India) for eight physico-chemical water quality parameters namely, pH, turbidity, chlorides, total hardness, nitrates, iron, residual chlorine and fluoride [9]. The second water sample was kept in a thermo-insulated icebox (4 °C) and was transferred to the microbiology laboratory for analysis of bacterial contamination.

### 2.2. Enumeration of Total Coliform and Identification of Escherichia coli

For estimation of total coliform count of bacteria in water samples, the Most Probable Number (MPN) method was used. Multiple tube technique was employed [10]. In brief, Mac-Conkey broth medium was used in 15 test tubes, in which inverted Durham tubes were added for gas collection. The observation on acid and gas production in each row of five tubes was noted and coliform count was recorded using McCrady’s table. *E. coli* was differentiated from other fecal coliforms using selective medium, eosin methylene blue agar (in this medium, the colonies of *E. coli* show typical metallic sheen with a dark center), followed by Eijkman test [11]. Identified *E. coli* isolates were further confirmed by the Vitek 2 (bioMerieux, Marcy l’Etoile, France) compact instrumentation [12].

### 2.3. Antibiotic Susceptibility

Confirmed *E. coli* isolates were tested for their antibiotic susceptibility by using the Vitek 2 instrument. Two cards, AST-GN25 and AST-EXN7 were selected, in which a total of 34 antibiotics were present for testing (Table A1). Antibiotics included were: tetracyclines, penicillins, all generation cephalosporins, carbapenems, trimethoprim/sulfamethoxazole and quinolones. Care was taken to include antibiotics that were used in the study area. The Vitek 2 instrument is an automated antibiotic susceptibility testing system that enables rapid determination of Minimum Inhibitory Concentrations (MICs) [13,14]. It uses an Advanced Expert System (AES) that provides standardized interpretative reading of MICs [15]. Updated AES parameters were set in the system to utilize default interpretation guidelines of the Clinical and Laboratory Standards Institute (CLSI) and natural resistance. Multi-Drug Resistance (MDR), defined as “resistance to three or more antibacterial classes” [16], was also evaluated.

### 2.4. Collection of Data on Occurrence of Diarrhoea and Antibiotic Use

The data on the occurrence of diarrhoeal cases was collected at regular intervals during the study period. Information was also collected on number of toilets from each household of the study villages once during the study period. Data on antibiotics delivered to sub-centres of primary health centres (PHCs) in the study villages from 2008–2012 was obtained from records. This data was used as proxy for antibiotics consumed by the population in the study villages and is presented as Defined Daily Doses (DDDs)/1,000 inhabitants/year. The DDD is the assumed average maintenance dose per day for a drug used for its main indication in adults [17].

### 2.5. Data Analysis

Data was entered in Excel version of MS-Office 2010 and analyzed using Stata 12.1 (Stata Corp., College Station, TX, USA). Descriptive statistics was used to present the data. Odds ratio (OR) was used to determine the strength of association between IWMP and coliform contamination of water. Pearson’s chi-square test was used to test statistical significance as applicable. A value of *p* < 0.05 was considered statistically significant while a *p*-value between 0.05 and 0.10 was considered as of “borderline significance”. A pilot study was conducted for one month in the study area before the main study.

### 2.6. Ethical Approval

Ethical approval was obtained from the institutional ethics committee of R. D. Gardi Medical College, Ujjain (No. 175/2011). Permission was also obtained from concerned local authorities for the collection of water samples and collection of data related to the study.

## 3. Results and Discussion

Out of the seven wells included in the study, one well from IWMV (IWMV4) was dry for three months, from April to June and one well from NWMV (NWMV2) was dry for five months, from February to June. During this period, the villagers used other wells in their respective areas, already included in the study. In total, 76 water samples, 45 from IWMV and 31 from NWMV, were collected over a period of one year.

Table 1 shows physico-chemical quality parameters of each well of the six tribal villages. The pH of all samples was in the range of 7.0 to 7.4. Turbidity in all samples was below 5 Nephelometric Turbidity Units (NTU) except for samples from the sources IWMV2 and NWMV2, which were in the range of 5–25 and 0–10 NTU respectively during the year. Chloride content was in the range of 20–60 ppm in IWMV and 20–30 ppm in NWMV. There was not much difference in total hardness of water in both the settings and it ranged between 50–200 ppm during the whole year. Nitrate content was below 10 ppm constantly throughout the year in all samples except IWMV1 and IWMV2, where a maximum value of up to 45 ppm was recorded in the months of June and July. There were no traces of iron, residual chlorine or fluoride in any of the samples.

**Table 1:** Physico-chemical quality parameters (average/month) of water from each well of six tribal villages with and without implementation of integrated watershed management programme.

Parameter	Integrated Watershed Management Villages (IWMV)				Non-Watershed Management Villages (NWMV)		
	Water Sources				Water Sources		
	IWMV1	IWMV2	IWMV3	IWMV4	NWMV1	NWMV2	NWMV3
	*n* = 12	*n* = 12	*n* = 12	*n* = 9	*n* = 12	*n* = 12	*n* = 7
pH	7.2	7.2	7.1	7.2	7.1	7.2	7.3
	(7.0–7.3)	(7.0–7.4)	(7.0–7.3)	(7.0–7.4)	(7.0–7.3)	(7.0–7.4)	(7.0–7.4)
Turbidity ^a^ (NTU)	2.5	13.7	0 *	0.6	1.3	3.3	1.3
	(0–5)	(5–25)		(0–5)	(0–5)	(0–10)	(0–5)
Chloride content ^b^ (ppm)	25	22.5	20.8	20 *	20.8	20 *	20 *
	(20–60)	(20–40)	(20–30)		(20–30)		
Total hardness ^c^ (ppm)	98	106	79	83.3	54	56	81
	(50–125)	(75–200)	(50–175)	(75–125)	(50–100)	(50–175)	(50–150)
Nitrate content ^d^ (ppm)	15.8	12.3	10 *	10 *	10 *	10 *	10 *
	(10–45)	(10–45)					

Notes: *n*—Number of samples; According to World Health Organisation (WHO) [18]—Maximum Acceptable Limits (MAcL) and Maximum Allowable Limit (MAlL) are; ^a^ MAcL = 5 NTU, MAlL = 25 NTU; ^b^ MAcL = 200 ppm, MAlL = 600 ppm; ^c^ MAcL = 50 ppm, MAlL = 200 ppm; ^d^ MAcL = 10 ppm, MAlL = 45 ppm; figures in parenthesis indicate range. * The asterix indicates that there was no variation between the samples. All values are rounded off to the nearest decimal point.

Quality of water has always an impact on human health. In general, lack of infrastructure for safe water facilities, makes tribal people prone to suffer health hazards due to water contaminants, both physico-chemical and microbiological [18]. The World Health Organization (WHO) has recommended levels for water quality parameters that are safe for human health. Two limits have been defined, safe acceptable limit (maximum concentration generally acceptable to consumers) and allowable acceptable limit (maximum concentration beyond which potability is seriously impaired) [19]. During our study, the physico-chemical water quality parameters in both IWMV and NWMV were within safe allowable limits for consumption. While the turbidity level was within acceptable limits (5 NTU) for other wells, it ranged up to 25 NTU in IWMV2. However, this turbidity level was also within allowable limit. IWMV2 was an old well, constructed prior to implementation of the IWMP, needing some repairs, but still used by the people. The turbidity that occurred in rainy season in this particular well might be due to leaching or surface runoff. This finding indicates that as part of the IWMP, attention should be paid to old wells which should be repaired to perfection.

The nitrate content was comparatively higher in IWMV during the rainy months of June and July. The implementation of IWMP results in increased agricultural activity. In the area of our study, the main agricultural activity occurred in the rainy season. During rainy season, leaching of substances occurs from farmland waste in groundwater through percolation and results in increased nitrate content [20,21]. The increased nitrate content in the IWMV wells might be due to the increased agricultural activity in IWMV. 

Table 2 shows the month-wise bacterial contamination level in each water source with coliform count/100 mL of water along with antibiotic resistance patters of the detected *E. coli*. In the case of IWMV, in 17 samples out of 45 (38%), coliform contamination was detected. This figure was 18 out of 31 (58%) in case of NWMV. The contamination level measured as coliform count per 100 mL was in the range of 0 to 7,900 for IWMV and 0 to 13,000 for NWMV. The odds of water samples to have coliform contamination was 2.3 times higher in NWMV as compared to IWMV (95% CI 0.81–6.45, *p* = 0.081). The P value for this difference showed borderline significance and a wide confidence interval indicated the results to be tilted towards IWMV suggesting a contribution of components of IWMP on reduction in bacterial contamination of water.

According to the recommendations by the WHO and Central Pollution Control Board of India, drinking water should be free from coliform contamination to be considered safe for use [19,22]. It has been observed that improper sanitation practices are responsible for bacterial contamination of water [2]. The results of coliform count in water from our study (Table 2) show that water from both IWMV and NWMV was contaminated on several occasions. However, the number of times contamination was detected and the count of coliform were lower in IWMV. Awareness about hygiene and improved sanitation practices due to the implementation of IWMP may be a contributory factor for the reduction in bacterial contamination of water in IWMV [23]. The number of households having toilets were significantly higher in IWMV (83/142, 58%) compared to NWMV (27/144, 19%), where open air defecation was the normal practice. In both types of villages, 85% of households had cattle. Animal litter can also be a potential source of water contamination in these villages, which may have resulted in the contamination of water in IWMV as well as in NWMV especially in the rainy season.

The study villages were located on hills. The wells were the only source of water in the villages. Because of the elevated location, the wells normally dried up in the summer season. One of the expected outcomes in villages with “integrated watershed management implementation” is increase in periods of water availability in wells and less dry periods compared to NWMV. It has earlier been found that water sources that become dry in the summer season remain more contaminated throughout the year [24]. Thus, due to integrated watershed management, in the implementation area, dry period for wells as well as contamination of their water would be expected to be low. Of the included water sources, one well out of three in NWMV and one well out of four in IWMV dried up during the dry season. NWMV3 remained dry for five months during the study period and was also found to be the most frequently contaminated water source (86% samples). As compared to this, IWMV3, which was dry for three months, was found to be less contaminated (56% samples). In several areas of the world, as in India, the tribal people reside in hilly terrains and forest areas. There they have to face severe water scarcity during summer season [24], with wells having scanty water reserve and hence contaminated water. Integrated watershed management aims to make water sources available that can supply water throughout the year. This could help to decrease the contamination of water as fewer sources dry up during the dry season. 

**Table 2:** Month-wise coliform count/100 mL water and antibiotic resistance of *Escherichia coli* isolated from water sources of tribal villages with and without implementation of integrated watershed management programme.

Month	Integrated Watershed Management Villages (IWMV)				Non-Watershed Management Villages (NWMV)		
	Coliform Count/100 mL				Coliform Count/100 mL		
	S/R (Resistance to Mentioned Antibiotics)				S/R (Resistance to Mentioned Antibiotics)		
	Water Sources				Water Sources		
	IWMV1	IWMV2	IWMV3	IWMV4	NWMV1	NWMV2	NWMV3
2011/07	170	7900	0	33	140	41	7000
	**R** (NA, TE)	**S**		**S**	**R** (CEP)	**S**	**S**
2011/08	14	0	0	0	7.8	7.8	0
	**R** (NA, TE)				**R** (NIT)	**S**	
2011/09	130	22	220	0	0	13	13000
	**R** (NA)	**R** (AMP, TI, PI)	**S**			**S**	**R** (AMP, TI, PI64, TE)
2011/10	350	240	0	350	0	79	920
	**S**	**S**		**S**		**S**	**S**
2011/11	0	7.8	280	140	0	11	42
		**S**	**R** (NA, TE, NIT)	**S**		**S**	**S**
2011/12	0	0	0	0	140	0	130
					**S**		**R** (NA, CEP)
2012/01	0	0	0	33	0	140	280
				**R** (CEP)		**S**	**S**
2012/02	350	0	0	140	0	350	a
	**S**			**S**		**R** (AMP, TI, PI, NA, TE, COT, NIT)	
2012/03	0	0	0	0	0	130	a
						**R** (AMP, TI, PI, NA, TE, COT, AT, All cephalosporins-ESBL)	
2012/04	94	0	0	a	0	0	a
	**S**						
2012/05	0	0	0	a	130	0	a
					**R** (CEP)		
2012/06	0	0	0	a	0	0	a
Frequency of contamination (%)	6/12	4/12	2/12	5/9	4/12	8/12	6/7
	(50)	(33)	(17)	(56)	(33)	(67)	(86)

Notes: N—Total number of samples; S—Susceptible; R—Resistant; 0—Absence of coliform; a—No sampling; NA—Nalidixic acid (MIC ≥ 32); TE—Tetracycline (MIC ≥ 16); AMP—Ampicillin (MIC ≥ 32); TI—Ticarcillin (MIC ≥ 128); PI—Piperacillin (MIC ≥ 128); PI64—Piperacillin (MIC ≥ 64); NIT—Nitrofurantoin (MIC = 64); CEP—Cephalothin (MIC = 64); COT—Co-trimoxazole (MIC ≥ 320); AT—Aztreonam (MIC = 4); All cephalosporins tested in two AST cards- [Cephalothin (MIC = 64); Cefazolin (MIC ≥ 64); Cefuroxime (MIC ≥ 64); Cefotetan (MIC = 2); Cefuroxime Axetil (MIC ≥ 64); Cefpodoxime (MIC ≥ 8); Cefotaxime (MIC ≥ 64); Ceftizoxime (MIC ≤ 1); Cefepime (MIC = 2)]; ESBL—Extended Spectrum Beta-Lactamases; Unit for MIC values is mg/L.

The proportion of inhabitants treated for diarrhoea at the PHC subcentres was significantly lower (*p* = 0.001) in the IWMV (18/663) compared to the NWMV (42/639). All the diarrhoeal cases in IWMV were in wet season while in NWMV, 23 cases were in wet season and the remaining 19 were in dry season. It is known that tribal people suffer from diarrhoea due to improper sanitation practices and contamination of water [18,25]. Drying of water sources is one of the responsible factors for contamination of water [24]. It has been stated earlier that due to implementation of IWMP, the number of households with toilets were higher in IWMV reducing their open air defecation practice. The wells of IWMV were also dry for lesser periods and less contaminated. Our earlier study in these villages showed that tribal people in the study setting perceived that implementation of IWMP improves the quantity and quality of water in water sources and they also perceived reduction in occurrences of diarrhoea due to it [23]. 

Total antibiotics delivered to PHC sub-centres in both types of villages are presented as DDDs per 1,000 inhabitants per year in Table 3. The figures were higher in IWMV as compared to NWMV during the study period. This was also true for the previous three years. Recent data on antibiotic dispensing in the community in European countries indicate the figures to be varying from 3,832 to 14,089 DDDs/1,000 inhabitants/year [26]. Population based data, to date, to our knowledge are not reported from India [27]. In our study villages, we could use the existing population as denominator. Antibiotic dispensing was low in our study villages, but between the two settings it was comparatively higher in IWMV.

**Table 3:** Data on yearly average antibiotics delivered (DDDs/1,000 inhabitants/year) to sub-centres of Primary Health Centre during the years 2008–2011 and 2011–2012 in tribal villages with and without the implementation of integrated watershed management programme.

Antibiotic, Assigned DDD	2008–2011 (June–May)		2011–2012 (June–May)	
	IWMV	NWMV	IWMV	NWMV
Tetracycline, 1 g	458	391	302	39
Ampicillin, 2 g	6	--	--	--
Amoxicillin, 1 g	163	33	38	58
Co-trimoxazole, 2 g	267	225	124	99
Erythromycin, 1 g	--	27	--	--
Ciprofloxacin, 1 g	7	52	--	--
Norfloxacin, 0.8 g	75	47	189	156
Nalidixic Acid, 4 g	--	--	1.5	--
Total DDDs/1,000 inhabitants/year	976	775	654	352

Notes: IWMV—Integrated Watershed Management Villages; NWMV—Non-Watershed Management Villages; DDD—Defined Daily Dose; PHC—Primary Health Center.

Based on our earlier study [23] the reasons for increased antibiotic dispensing in IWMV could be proposed as follows. An important outcome of implementation of IWMP is the socio-economic development that takes place in the target area. This results in increased health awareness and reduced migration. Thus, in the IWMV there is an increased awareness about utilizing health services which might have led to more antibiotic prescriptions than in NWMV. Further, because of less economic development, people from NWMV migrate more frequently for work outside their villages than people from IWMV, thus the population staying throughout the year in IWMV is higher as compared to NWMV. There are also differences in seeking traditional healing measures between the two settings. It was reported that the people from NWMV believed more in using traditional measures compared to people from IWMV [23]. All these factors might have resulted in more delivery of antibiotics in IWMV. 

Our findings showed that in the study area 63% (IWMV 65%, NWMV 61%) of *E. coli* isolates were susceptible to all tested antibiotics (Table 4). A recent study from Nigeria showed that all the 35 *E. coli* isolated from water samples from open dug wells were resistant to at least one antibiotic [28]. A study from India shows that 96% out of the 151 *E. coli* isolates from household drinking water (where tube wells and open wells were main sources of water) were resistant to at least one antibiotic [29]. A study conducted on water samples from hilly areas of Uttarakhand State, India, showed that all the 20 *E. coli* isolates were resistant to at least one antibiotic and 90% of isolates were MDR [30]. A study from Pakistan showed that of the 27 *E. coli* isolated from drinking water samples 93% were resistant to one antibiotic and 63% of the isolates were MDR [31]. A study from China showed that out of 114 *E. coli* isolated from surface (lake) water samples, 85% were resistant to one antibiotic [32]. In these five references analyzing susceptibility of *E. coli* isolates from water samples, the overall susceptibility indicated was 0%, 4%, 10%, 7% and 15% respectively, very low figures compared to the overall 63% susceptibility in our *E. coli* isolates.

**Table 4:** Co-resistance and Multi Drug Resistance (MDR) of *Escherichia coli* from well water of tribal villages with and without the implementation of integrated watershed management programme.

Resistance	IWMV (*N* = 17)	NWMV (*N* = 18)
	*n* (%)	*n* (%)
Resistant to one group of antibiotics	3 (18)	3 (17)
Resistant to two groups of antibiotics (co-resistance)	2 (11)	2 (11)
Resistant to three or more than three groups of antibiotics (MDR)	1 (6)	2 (11)
Susceptible to all	11 (65)	11 (61)

Notes: *N*—Total number of isolates; IWMV—Integrated watershed management villages; NWMV—Non-watershed management villages.

The number of samples containing antibiotic resistant *E. coli* were similar between the two types of villages, with 6/17 in IWMV and 7/18 in NWMV. Amongst the resistant *E. coli* isolates three isolates from both the settings *i.e.*, IWMV and NWMV were resistant to only one group of antibiotics (Table 4). Two isolates from NWMV were MDR compared to a single MDR isolate in IWMV. In comparison with other studies, MDR was low in the *E. coli* isolated from the water sources of the study villages [30,31]. There was one isolate from NWMV which produced ESBL (Extended Spectrum Beta-Lactamases) (Table 2). 

The isolates listed in Table 5 are representative *E. coli* isolates from each of the 17 contamination events observed in the IWMV wells and 18 contamination events observed in the NWMV wells. Table 5 shows the antibiotic resistance of these *E. coli* isolates to antibiotics of different groups. Resistance to tetracycline and nalidixic acid was most common in both the settings. Nalidixic acid resistance is the first step towards the quinolone resistance. High frequency of resistance to tetracycline and quinolone was also recorded in another part of India [29]. The resistance to tetracycline and quinolones has been explained as due to their lower degradation rates (half-life 100 days) in the environment [33]. The longer exposure of bacteria to antibiotic residues in aquatic environment creates a selective pressure and increases the risk of development of resistance [34,35]. 

**Table 5:** Antibiotic resistance of *Escherichia coli* isolated from well water in tribal villages with and without the implementation of integrated watershed management programme

Antibiotics	IWMV (*N*=17) *n*	NWMV (*N*=18) *n*
Tetracycline	3	3
Ampicillin	1	3
Cephalothin (1st)	1	4
Cefuroxime (2nd)	0	1
Cefotaxime (3rd)	0	1
Cefepime (4th)	0	0
Aztreonam	0	1
Meropenam	0	0
Co-trimoxazole	0	2
Amikacin	0	0
Nalidixic acid	4	3
Nitrofurantoin	1	2

Notes: IWMV—Integrated Watershed Management Villages; NWMV—Non-Watershed Management Villages; *N*—Total number of isolates; *n*—Resistant isolates; 1st—1st generation cephalosporins; 2nd—2nd generation cephalosporins; 3rd—3rd generation cephalnosporins; 4th—4th generation cephalosporins.

Table 5 shows that *E. coli* isolates from NWMV exhibited resistance to wider range of antibiotics. Resistance was recorded to 2nd and 3rd generation cephalosporins, nitrofurantoin and aztreonam in NWMV, which were not delivered in these villages. Co-trimoxazole, which contains trimethoprim, was the second most delivered antibiotic in these villages. In an earlier study, it was found that there is a cross-correlation between usage of one antibiotic and resistance to another, particularly usage of trimethoprim and resistance to ampicillin and usage of trimethoprim and resistance to cephalosporins [36]. Plasmid mediated resistance and horizontal transfer of resistance between bacteria have also been implicated for transfer of resistance [37,38]. It is difficult to explain the observed resistance to nitrofurantoin and aztreonam, however, the phenomenon of development of cross-resistance is not fully understood. Seasonal migration in tribal areas is also reported to be responsible for many public health issues [23]. If migration has any role in this, in terms of carrying resistant bacteria from migratory sites to the respective native villages, then an integrated watershed management policy which might be helpful in reduction of migration by generation of employment opportunities in tribal areas might thus become also helpful in curtailing the spread of antibiotic resistance. As per our knowledge, this is the first study on contamination of water and occurrence of antibiotic resistance in *E. coli* from water sources in hilly tribal areas, in the context of watershed management. The strength of this study is that all the water sources in the included tribal villages were followed for a whole year. Secondly, we maintained as much similarity as possible with respect to geographical and social patterns between the villages by selecting villages in a short radial distance. There were also some limitations of this study. As integrated watershed management was the criterion behind selection of villages and it was necessary to choose villages in the same geographical locality for appropriate comparison, there were very few integrated watershed management villages available for the study. That was the reason for the relatively small sample size. In the study villages, all the people generally relied on PHC sub-centres for health services including medicines, so we cannot exclude that people at some occasions might have received medicines from other sources. Seasonal migration for employment by the people, particularly in NWMV may also have affected some parameters of the study as explained above.

## 4. Conclusions

This study found that amongst the included villages, bacterial contamination of water and diarrhoeal cases in the community were lower in villages in which integrated watershed management was implemented compared to villages where it had not been implemented. *E. coli* isolates from non-watershed management villages showed resistance to a wider range of antibiotics. Collectively, there was a high level of susceptibility (63%) to antibiotics in the analyzed *E. coli* isolates from the water sources of the hilly tribal villages in study area. This information is valuable in the context of reports of high antibiotic resistance all over the world. 

## Appendix

**Table A1:** List of antibiotics used for susceptibility testing using the Vitek 2 AST (antibiotic susceptibility test) cards.

Card No.-	AST GN 25	Card No.-	AST EXN7
Sr. No	Antimicrobial	Sr. No	Antimicrobial
1	ESBL	1	Amoxicillin/Clavulanic acid
2	Ampicillin	2	Ticarcillin
3	Ampicillin/Sulbactum	3	Ticarcillin/Clavulanic acid
4	Piperacillin/Tazobactum	4	Piperacillin
5	Cefazolin	5	Cephalothin
6	Cefriaxone	6	Cefuroxime
7	Cefepime	7	Cefuroxime Axetil
8	Aztreonam	8	Cefotetan
10	Imipenam	10	Cefotaxime
11	Meropenem	11	Ceftizoxime
12	Amikacin	12	Aztreonam
13	Gentamicin	13	Meropenem
14	Tobramicin	14	Nalidixic Acid
15	Ciprofloxacin	15	Moxifloxacin
16	Moxifloxacin	16	Norfloxacin
17	Tigecycline	17	Tetracycline
18	Nitrofurantoin	18	Tigecycline
19	Trimethoprim/ Sulfamethoxazole		
